# Current Insights into Industrial Trans Fatty Acids Legal Frameworks and Health Challenges in the European Union and Spain

**DOI:** 10.3390/foods13233845

**Published:** 2024-11-28

**Authors:** Pablo Javier Miró-Colmenárez, Esther Illán-Marcos, Eliana Díaz-Cruces, María Méndez Rocasolano, José Manuel Martínez-Hernandez, Ezequiel Zamora-Ledezma, Camilo Zamora-Ledezma

**Affiliations:** 1 Law Ecotechnology and Innovation Keys for the 21st Century Development Research Group, Faculty of Law, UCAM-Universidad Católica San Antonio de Murcia, Campus de los Jerónimos 135, Guadalupe, 30107 Murcia, Spain or esther.illanmarcos@gmail.com (E.I.-M.); or fediaz@alu.ucam.edu (E.D.-C.); mmrocasolano@ucam.edu (M.M.R.); 2 Department of Nutrition and Food Technology, UCAM-Universidad Católica San Antonio de Murcia, Campus de los Jerónimos 135, Guadalupe, 30107 Murcia, Spain; jmmartinez3@ucam.edu; 3 Ecosystem Functioning & Climate Change Team—FAGROCLIM, Faculty of Agriculture Engineering, Universidad Técnica de Manabí (UTM), Lodana 13132, Ecuador; ezequiel.zamora@utm.edu.ec; 4 Higher Polytechnic School, UAX-Universidad Alfonso X el Sabio, Avda. Universidad, 1, Villanueva de la Cañada, 28691 Madrid, Spain; 5 Green and Innovative Technologies for Food, Environment and Bioengineering Research Group (FEnBeT), Faculty of Pharmacy and Nutrition, UCAM-Universidad Católica San Antonio de Murcia, Campus de los Jerónimos 135, Guadalupe, 30107 Murcia, Spain

**Keywords:** industrial trans-fatty acids, artificial trans-fatty acids, regulations, human health, ultra-processed food

## Abstract

The presence of industrial trans-fatty acids (iTFAs) in processed foods poses significant public health concerns, necessitating comprehensive regulatory frameworks. In this study, the current legal landscape governing iTFA in the European Union and Spain is analyzed, with a particular focus on regulatory effectiveness and implementation challenges. The research methodology combines a systematic review of existing regulations, including EU Regulation No. 1169/2011 and Spanish Law 17/2011, with the analysis of the scientific literature on iTFA health impacts. The results reveal significant regulatory gaps, particularly in enforcement mechanisms and iTFA detection methods. Key challenges are also identified in the present study, including inconsistent compliance monitoring, varying analytical methods for iTFA detection, and contradictions between EU and Spanish regulatory frameworks. Additionally, in this work, the need for harmonized approaches to ultra-processed food regulation is emphasized. Further, the conclusion is that despite the current regulations providing a foundation for iTFA control, it is compulsory to enhance the monitoring systems, and clearer regulatory guidelines are necessary. These would contribute valuable insights for policymakers, food industry stakeholders, and public health professionals working towards effective iTFA regulation.

## 1. Introduction

**Figure 1:**
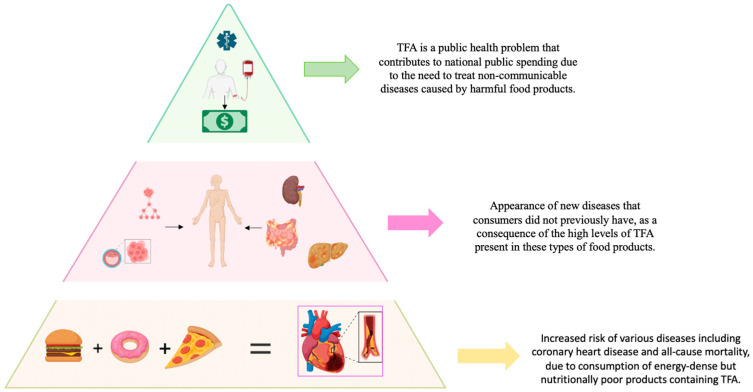
A schematization of the human health impact of TFAs.

The consumption of foods with industrial trans-fatty acids (iTFAs) is associated with multiple health risks. The main effects include cardiovascular diseases (increasing death risk by 28%), type 2 diabetes, systemic inflammation, and certain types of cancer. A 2% increase in daily iTFA intake raises cardiovascular disease risk by 23% [1,2,3,4,5,6,7,8,9,10]. Recent studies in Europe have found that some fast-food products contain more than 50% of the WHO recommended daily limit [3,6], which has led to the implementation of strict regulations in the EU, such as Regulation 2019/649, to minimize their presence in food [11]. Figure 1 show a schematization of the human health impacts of iTFAs.

In this context, the high mortality rates from non-communicable diseases (NCDs) caused by the consumption of unhealthy food products are a global concern. Indeed, there is a link between low economic status and access to nutritionally poor-quality food products. Consequently, one of the most effective measures to promote a healthier population is through robust legislation that limits the use of synthetic, unhealthy components in food production. To ensure that food products retain their natural properties and provide health benefits to consumers, governments should reenforce their regulations or prohibit certain industrial practices that lead to excessive and unnecessary modifications. It is important to note that the right to health is a fundamental human right directly related to the right to life. Therefore, if governments fail to legislate effectively on factors affecting health, such as food and nutrition, they are essentially violating the right to life of their citizens. Moreover, governments must actively fulfill their duty to respect and protect the basic needs that constitute the human and fundamental rights of their citizens. Thus, it is mandatory to address critical gaps in the current research regarding iTFA regulation and health impacts in the EU and Spain. Despite the existing literature having documented the health risks of iTFA consumption, there remains limited analysis of the effectiveness and implementation challenges of current regulatory frameworks

Therefore, considering the negative health impacts of iTFAs in food products and the urgent need for effective and updated regulations, in this work, the current legal landscape is analyzed governing iTFAs in the European Union and Spain, with a particular focus on regulatory effectiveness and implementation challenges. The research methodology combines a systematic review of the existing regulations, including EU Regulation No. 1169/2011 and Spanish Law 17/2011, with an analysis of the scientific literature on iTFA health impacts. The results reveal significant regulatory gaps, particularly in enforcement mechanisms and iTFA detection methods. In the present study, key challenges are also identified, including inconsistent compliance monitoring, varying analytical methods for iTFA detection, and contradictions between EU and Spanish regulatory frameworks. Additionally, in this work, the need is emphasized for harmonized approaches to ultra-processed food regulation. Further, in this study, the conclusion is that despite the current regulations providing a foundation for iTFA control, it is compulsory to enhance the monitoring systems, and clearer regulatory guidelines are necessary. These would contribute valuable insights for policymakers, food industry stakeholders, and public health professionals working towards effective iTFA regulation.

## 2. Methodology

This literature review was conducted using ScienceDirect and Scopus databases to analyze the EU and Spain legal frameworks regarding industrial trans fats, ultra-processed food products, and related regulations. This review followed the PRISMA guidelines for systematic reviews. Indeed, the search strategy and selection process were conducted using the following Boolean combinations:

(a) “EU legal framework” AND (“industrial trans fats” OR “trans fatty acids”);
(b) “Spain legal framework” AND (“industrial trans fats” OR “trans fatty acids”);
(c) “Ultra-processed food products” AND (“regulation” OR “trans fats”);
(d) “Trans fats regulation” AND (“European Union” OR “Spain”).

Further, inclusion and exclusion criteria were employed to provide reliable boundaries of the present study, as follows.

(a) Inclusion: articles published between 2010 and 2024, English/Spanish language, peer-reviewed;
(b) Exclusion: non-peer reviewed articles, duplicates, irrelevant content.

The initial search yielded 487 articles. After removing duplicates and applying inclusion/exclusion criteria, 156 articles were selected for full-text review. The final analysis included 98 articles. In Table 1, the distribution of the most relevant reviewed literature by subject area is shown. For its part, in Table 1, the most relevant (the top five) contributing journals are shown.

After that, all the data obtained were systematically extracted using a standardized form capturing the most relevant information including but not limited to the following:

(a) study design and methodology;
(b) sample size and characteristics;
(c) key findings and conclusions;
(d) regulatory implications;
(e) health impact assessments.

Further, the extracted data were analyzed thematically to identify patterns and trends as follows:

(a) regulatory approaches across jurisdictions;
(b) health impact evidence;
(c) implementation challenges;
(d) industry compliance measures.

Additionally, relevant regulations and legislation were examined, including EU Regulation 2019/649, Law 17/2011, and Directive 1999/21/EC, to provide comprehensive coverage of the legal framework. This enhanced methodology provides a more transparent and rigorous foundation for our analysis of iTFA regulation and its implications.

## 3. State of the Art: Key Concepts

### 3.1. Definition and Types of Industrial Trans-Fatty Acids (iTFAs)

Industrial trans-fatty acids (iTFAs) are a type of fat that are created by partially hydrogenating vegetable oils. They are commonly found in processed and fried foods, and have been linked to various health problems, such as heart disease and type 2 diabetes [12,13]. While fats are a necessary macronutrient for the body, not all fats are created equal. There are different types of fats, including saturated, unsaturated, and *trans fats*. Saturated fats are typically found naturally in animal products and have been linked to an increased risk of heart disease [14]. Unsaturated fats, on the other hand, can be further divided into monounsaturated and polyunsaturated fats, and are generally considered to be healthier than saturated fats [15]. Industrial trans-fatty acids (iTFAs), however, are widely considered to be the least healthy type of fat, and have been limited or prohibited in many countries. In this context, the World Health Organization (WHO) recommends that *trans-fat* intake be limited to less than 1% of total energy intake, as even small amounts can increase the risk of heart disease [16,17].

### 3.2. Trans-Fatty Acids

Trans-fatty acids are a specific type of fat found in some foods. They have a unique shape at the molecular level, which affects how our bodies process them [18]. While small amounts occur naturally in some animal products, most *trans fats* we encounter are made in factories. The WHO definition stablished that *trans fats* are “unsaturated fatty acids with at least one double bond in the trans configuration” [19]. Food companies create them by adding hydrogen to liquid vegetable oils, making them more solid and less likely to spoil.

This process helps processed foods last longer. Common foods that often contain these manufactured *trans fats* include certain margarines, packaged snacks, baked goods, and foods cooked in partially hydrogenated oils. Health experts have found that eating too much of these artificial *trans fats* can be bad for our hearts. They can raise the levels of “bad” cholesterol in our blood while lowering the “good” cholesterol, which may increase the risk of heart problems and other health issues. Indeed, human beings require a balanced diet to provide the necessary nutrients for carrying out vital functions [20]. Nutrients are essential elements that can be used as energy, structural material, or as control agents for the organism’s reactions, and they can be found in food [21]. Macronutrients, such as proteins, carbohydrates, and fats, are needed in large amounts, while micronutrients, such as vitamins and minerals, are needed in smaller amounts [22]. Fats, also known as lipids, are a crucial macronutrient in the diet.

They are mixtures of triglycerides made up of different types of fatty acids and serve as a source of energy for the body [23]. Additionally, they are necessary for the absorption of certain vitamins and minerals and play a vital role in the construction of cell membranes, blood clotting, and muscle inflammation [24]. However, not all fats are created equal, and some can be harmful to health [25]. Indeed, consuming *trans fats*, particularly industrial *trans fats*, has been associated with an increased risk of developing metabolic disorders and chronic diseases [26]. Industrial *trans fats* are artificially produced during the hydrogenation process of vegetable oils, which increases their shelf life and enhances their flavor [27]. Interestingly, several studies have shown that replacing industrial *trans fats* with unsaturated fats can reduce the risk of developing these metabolic disorders [28].

On the other hand, unsaturated fats, typically found naturally in plant-based products, are considered healthier than saturated fats and have been shown to have beneficial effects on heart health [29]. In contrast to *trans fats*, which are often found in highly processed foods, and associated to an increased risk of several diseases [30], unsaturated fats such as omega-3 fatty acids, found in fatty fish and certain plant-based foods, have been associated with reduced inflammation and a lower risk of chronic diseases such as heart disease and arthritis [31]. To better understand this, it is worth mentioning that during the nutrition process, our body assimilates all types of fats found in foods or products ingested. Both beneficial and harmful fats are assimilated because nutrition is involuntary, meaning that once people select and ingest certain foods, a set of involuntary processes occur through which both substances are absorbed [20]. However, to maintain a healthy metabolism, a perfect balance of TFA absorbed by the organism should be achieved.

### 3.3. Main Types of Fats in Foods

Understanding the main differences between the different types of fats found in food and food products is crucial for maintaining good health [15]. *Trans fats* can be categorized into two main types, those that are naturally occurring (nTFAs) and artificial *trans fats* (iTFAs). Thus, nTFAs are found in small amounts in meat and dairy products from ruminant animals like cows, sheep, and goats. These fats are produced by bacteria in the stomachs of these animals and include examples such as conjugated linoleic acid (CLA) and vaccenic acid. Generally, nTFAs are considered safe when consumed in moderation. On the other hand, artificial *trans fats* are found in processed foods, such as margarine, snack foods, baked goods, and fried foods [32].

These fats are created through an industrial process called partial hydrogenation, where hydrogen is added to liquid vegetable oils to make them more solid. iTFAs are known to increase the risk of several diseases. Due to their harmful health impacts, these fats have been restricted in many countries. On the one hand, unsaturated fats (UFs), including monounsaturated and polyunsaturated fats, have been shown to have a positive impact on health when consumed in moderation, as for nTFAs [27,33]. On the other hand, saturated and TFAs have been linked to an increased risk of heart disease and other health problems [34]. By knowing the types of fats in the foods that people eat can help them make healthier choices and manage people’s diet and weight [35]. For example, foods that are high in unsaturated fats, such as avocados, nuts, and oily fish, can be a healthier choice than foods that are high in saturated and trans fats, such as red meat, fried foods, and baked goods [36,37]. Further, the content of TFAs in meat and milk products ranges from 3% to 6% [38]. TFAs can also be unintentionally created during oil refining procedures and food production, meaning that foods containing refined oils often contain small amounts of TFAs [39]. Several studies has shown that people who consume diets with a high content of unsaturated fats have a trend to lower body weights compared to those who consume diets rich in saturated and trans fats [40,41]. Furthermore, adequate food choices would impact significantly on the risk of chronic illnesses [41].

Measuring trans-fatty acids (TFAs) in foods also presents several challenges due to their diverse sources and the complexity of their analysis [42,43]. The accurate quantification of TFAs requires sophisticated analytical techniques like gas chromatography (GC) and Fourier transform infrared (FTIR) spectroscopy, which can be costly and require specialized knowledge. Additionally, the rapid reformulation of food products to reduce TFA content complicates the maintenance of up-to-date consumption data. There is also a lack of standardized protocols and regulatory frameworks in many regions, particularly in low- and middle-income countries, further hindering the consistent and reliable TFA monitoring. One of the most used methods to extract fat from foods is based on ultrasonic fat extraction (UFE). Typically, a few grams of the food sample are mixed with a solvent (hexane in a 1:10 ratio). To guarantee reproducibility, extraction is carried out at least in triplicate. The sample is then left for one hour prior to being subjected to ultrasound at 25 °C for 30 min. Further, the samples are centrifuged and the supernatant is evaporated, dried, and weighed. In order to measure the TFAs by chromatography, it is necessary to methylate these fats. The dried TFAs are then mixed again with hexane and a potassium hydroxide solution in methanol, and subsequently centrifuged. The supernatant contains the methylated esters. Finally, these samples are typically measured following the ISO 12966 method, which describes the procedures for the preparation and analysis of fatty acid methyl esters (FAMEs) using gas chromatography (GC). This standard is primarily used in the oil and fat industry to determine the fatty acid composition in various matrices, such as vegetable oils, animal fats, and derived products. Furthermore, this method allows the precise and reproducible determination of the fatty acid composition in oils and fats, being a fundamental tool in quality control and research in the food and oilseed product industry [44]).

### 3.4. Saturated Fatty Acids (sFAs)

Saturated fatty acids (sFAs) are long-chain carboxylic acids that contain no double bonds between the carbon atoms in their hydrocarbon chains, meaning they are “saturated” with hydrogen atoms. This lack of double bonds results in a straight, linear structure, which allows the molecules to pack closely together, making them solid at room temperature [43]. Saturated fats, such as lauric, myristic, palmitic, and stearic acid, are solid at room temperature and have a high melting point [45]. sFAs are primarily found in foods of animal origin such as cheese or red meat, but they can also be found in some plant foods such as coconut oil or palm oil. Currently, many industrial food products contain high amounts of saturated fatty acids (sFAs). Additionally, a high intake of such fats would increase the risk of developing various health problems [15,46]. Thus, choosing foods that are low in saturated fat and *trans fat* would be an essential step towards a healthy diet. Additionally, minimizing the consumption of ultra-processed foods, which are often high in saturated and *trans fats*, is also crucial for promoting good health [47].

### 3.5. Unsaturated Fatty Acids (uFAs)

Unsaturated fatty acids are a long-chain carboxylic acids characterized by one or more double bonds between carbon atoms in their hydrocarbon chains [48]. When two adjacent carbon atoms form a double bond instead of bonding to a hydrogen atom, an unsaturated bond is formed, giving this type of fat its name. The presence of double bonds causes unsaturated fats to have a lower melting point, making them liquid at room temperature. This type of fat is commonly found in plant-based foods such as nuts, seeds, avocados, and vegetable oils, as well as in fatty fish like salmon and mackerel. Generally, uFAs are considered healthier options for food preparations. Indeed, monounsaturated fatty acids (MUFAs) and polyunsaturated fatty acids (PUFAs) are two types of unsaturated fats. MUFAs can be found in foods such as olive oil, avocado, and nuts, while PUFAs are commonly found in fatty fish, vegetable oils, and nuts. These types of fats have been associated with a positive impact on health, decreasing the risk of developing CVD, type 2 diabetes, and certain types of cancer [15,49]. Unsaturated fats are divided into two subcategories based on their chemical structure, of monounsaturated fats (MUFAs) and polyunsaturated fats (PUFAs). MUFAs have one double bond in their structure, while PUFAs have two or more double bonds. Both types of fats are liquid at room temperature, but MUFAs are more resistant to oxidation than PUFAs. An example of MUFAs is olive oil, while an example of PUFAs is corn oil. Several studies have shown that consuming unsaturated fats can have health benefits [48,50]. PUFA consumption has been associated with improved cholesterol levels in the blood, including a reduction in the ratio of total cholesterol to high-density lipoprotein cholesterol (TC:HDL-C), which is a known predictor of cardiovascular disease risk [51]. Additionally, both MUFA and PUFA consumption have been associated with improved insulin sensitivity and reduced inflammation in the arteries [52,53]. Unsaturated fats also include omega-3 and omega-6 fatty acids, which are essential fatty acids that cannot be produced by the body and must be obtained through diet. Omega-3 fatty acids are found in fatty fish, flaxseeds, and walnuts, while omega-6 fatty acids are found in vegetable oils, nuts, and seeds. Both types of fatty acids play important roles in human health, including reducing inflammation and supporting brain and heart health [54]. Thus, incorporating unsaturated fats into the diet can have health benefits, including improved cholesterol levels, insulin sensitivity, and reduced inflammation. Consuming a variety of plant-based foods and fatty fish can help ensure an adequate intake of these important nutrients.

Although both sFAs and TFAs are solid or semi-solid at room temperature, the main difference between them is that sFAs have a natural origin, while most TFAs are considered synthetic because they are made by humans with the sole objective of reducing the production costs of food products or improving its sensorial properties [27]. In this context, many institutions, such as the Harvard Medical School, and countries have taken a position against the use of TFAs and have advocated for their removal from food production [55]. They have publicly stated that TFAs have no known health benefits and that there is no safe level of consumption. As a result, the United States has high restrictions on using TFAs in the food industry. Given the lack of health benefits associated with TFAs, there has been a growing interest in the use of healthier alternatives, such as MUFAs/PUFAs, as well as natural oils like olive and canola oil [56]. On these grounds, it seem essential to recognize the negative health implications associated with the consumption of TFAs and the importance of replacing them with healthier alternatives.

### 3.6. Ultra-Processed Food Products

Ultra-processed food products are industrially manufactured foods made from multiple ingredients, including substances not commonly used in home cooking, such as high-fructose corn syrup, hydrogenated oils, and protein isolates. These foods undergo various industrial processes like extrusion, molding, and pre-frying, and often include additives such as artificial flavors, colors, emulsifiers, and preservatives to enhance the taste, texture, and shelf life. Examples include but are not limited to soft drinks, packaged snacks, instant noodles, and reconstituted meat products. Such food products are typically high in sugar, fat, and salt, and are designed to be convenient, highly palatable, and profitable, often at the expense of nutritional quality [47]. In this respect, and according to a report from the WHO, the consumption of ultra-processed foods has been steadily increasing worldwide, especially within the European Union (EU), over the last decade. The report highlights that ultra-processed foods would reach up to 50% of the total daily energy intake in some European countries, including Spain [47,57]. In Spain, the consumption of ultra-processed foods has also increased significantly in recent years. A study by Moubarac et al. found that the consumption of ultra-processed foods increased from 20% of total energy intake in 2000 to 34% in 2012 among Spanish adults. The authors further claimed that a higher consumption of ultra-processed foods was associated with a higher risk of obesity and metabolic syndrome [58]. Similarly, another study analyzed the trends in ultra-processed food purchases in Spain from 2008 to 2018, and found that the proportion of ultra-processed food purchases increased from 23% in 2008 to 28% in 2018, with the highest increase observed in processed meat, sugary drinks, and snacks [19,59]. Thus, as observed from the literature, it seem that the consumption of ultra-processed foods has been steadily increasing worldwide, in the EU, and in Spain accordingly over the last decade, with all the potential negative impacts on health.

### 3.7. Trans Fatty Acids (TFAs) in the Food Industry

Ultra-processed products in the food industry require prolonged durability to extend their consumption date [4,43]. Therefore, the industry is constantly exploring techniques and synthetic compounds to create long-lasting food products, often compromising their nutritional quality. Table 2 lists the most consumed ultra-processed foods (UPFs) and the type of fats in the EU and Spain.

One of the most commonly used methods consists of the hydrogenation of fats, which has been used since the 1930s to save on production costs and obtain more stable products [12,43]. This process involves introducing hydrogen gas into vegetable oils, resulting in a solid or semi-solid substance with less susceptibility to oxidation than unsaturated fats. Partial hydrogenation saturates the double bonds incompletely, allowing the bonds to migrate to different positions or transform their configuration from cis to trans [60]. However, partially hydrogenated oils (PHOs) contain approximately 10% to 60% of *trans fats* [45], which have been also linked to harmful effects on human health [61]. Complete hydrogenation results in a product with 100% saturated fatty acids and which is believed to be free of *trans fats* [45]. Nevertheless, often residual *trans fats* may still be present in small percentages when oils and fats are modified or processed [60]. Partially hydrogenated oils are widely used in the food industry for baking products, such as industrial pastries, margarine, or its derivatives for frying. At present, food products should either state that they do not contain *trans fats* or contain less than 1% (according to regulations). However, these products may have high levels of saturated fats, which can also cause or trigger health problems related to blood pressure or blood flow [62].

**Table 2:** Most consumed ultra-processed foods (UPFs) and their type of fats in EU and Spain, adapted from [42,43,51,63,64].

Ultra-Processed Food Product	Type of Fats
*Potato Chips*	*Saturated Fatty Acids*
*Cookies*	*Saturated Fatty Acids, Unsaturated Fats*
*Margarine*	*Saturated Fatty Acids, Trans Fats*
*Bakery Products*	*Saturated Fatty Acids, Unsaturated Fats*
*Fast Food*	*Saturated Fatty Acids, Trans Fats*
*Processed Meats*	*Saturated Fatty Acids, Unsaturated Fats*

The food industry’s current alternatives to reduce the iTFA content include the use of trans-free margarines and products recognized as virtually trans-free (VTF) [65]. Additionally, they are required to use oils with a high degree of saturation, such as palm oil, to replace *trans fats* [66]. Many industries have incorporated the complete hydrogenation within their processing to reach 100% saturation, which is also known as a good option for replacing *trans fats* [66]. However, the drawbacks of these alternatives are that they may negatively affect health because the oil structure would be affected [67]. In this respect, according to the WHO and the Food and Agriculture Organization of the United Nations (FAO), the total fat intake for better health outcomes should be limited to 30%, with less than 10% coming from saturated fatty acids, 6-10% from PUFAs, and the rest from MUFAs [61]. It is worth mentioning that small amounts of TFAs can unintentionally be created during cooking and baking at high temperatures (over 180 degrees Celsius). Further, refined vegetable oils may also contain small percentages of *trans fats* [61]. Thus, it is essential to note that when reading food labels, consumers should be aware that the use of the term “refined vegetable oils” without specifying the exact vegetable used or mixture of oils may indicate the presence of TFAs resulting from the refining process [68].

### 3.8. Effects of Daily Consumption of Products Containing Industrial Trans Fats

*Trans fats*, prevalent in many processed foods and some animal products, are associated with significant health risks. Recognizing these dangers, health authorities like the WHO advise minimizing *trans-fat* intake and substituting them with healthier fats to enhance overall health and reduce the risk of chronic diseases [69,70]. In this context, partially hydrogenated trans-fatty acids (phTFAs) are considered a major risk factor for the development of cardiovascular diseases (CVDs) and should be avoided [71]. Indeed, CVDs remain the leading cause of death worldwide, among the entire population, regardless of age, gender, race, or nationality, accounting for 31% of global mortality in 2019 [72]. Mozaffarian et al. reported that the risk of dying from a heart attack is 21% higher when 2% of daily energy intake comes from *trans fats* [12]. Additionally, TFAs can cause an inflammatory response by modifying cytokine concentrations, which can further increase the risk of CVDs [73]. Further, TFAs can cross the placental barrier and potentially cause negative health outcomes during fetal development [12]. Similarly, several studies have demonstrated that TFAs increase the concentration of low-density lipoprotein (LDL) cholesterol, which is harmful, and decrease the concentration of high-density lipoprotein (HDL) cholesterol, which is beneficial, to a much greater extent than saturated fat [27,69,70]. There is also evidence linking the consumption of TFAs to an increased risk of type 2 diabetes [28] and certain types of cancer, including breast and ovarian cancer [74]. In this respect, the United Nations, for example, has recognized the significant number of deaths from coronary heart disease attributable to the consumption of TFAs and included a commitment to reduce premature deaths from nontransmissible diseases by 30% by 2030 in the Sustainable Development Goals (SDGs) [72]. This was underlined by targets 3.4 and 9 of the SDGs—2030 Agenda, in which the priority was established to reduce premature deaths including those caused by foods [39].

Another important issue often underestimated is the bioaccumulation of fatty acids. Indeed, TFAs can be accumulated in the human body by integrating into cell membranes and adipose tissue after ingestion. While TFAs do not persist indefinitely like some environmental toxins, their presence and negative effects can continue as long as they are consumed. The World Health Organization (WHO) recommends that *trans-fat* intake should be kept below 1% of total daily calories to mitigate the risk of cardiovascular diseases and other health issues. For instance, in a 2000-calorie diet, this equates to less than 2 g of *trans fats* per day. To illustrate, if a food product contains 0.25% *trans fats*, an individual could consume up to 800 g of that food without exceeding the WHO’s recommended limit. Adhering to these guidelines is crucial for minimizing the harmful effects of *trans fats* and promoting long-term health [69,70,75].

## 4. General Considerations About the Products That Contain TFAs

To date, there is a worldwide trend for a progressive decrease in the use and consumption of TFAs. However, vulnerable groups in developed countries more often consume unhealthy products with a high TFA content, among other reasons, due to their accessibility and the affordable price for these food products [76]. Typically, lower priced products are more susceptible to containing higher levels of TFAs as a consequence of some industrial processing techniques, so the harmful dietary effects on health would have a greater effect on the population with fewer economic resources. Additionally, determining the actual total intake of TFAs by individuals or groups is challenging, as the amount of TFAs in the same food product, even of the same brand, varies from one country to another. This can result in different data regarding the TFA content in the same product of the same brand, making it impossible to generalize about the same group of food products (Judd, 2015). For instance, the TFAs in meat derivatives depend on the animal from which they come [77]. Also, higher amounts of TFAs have been found in chicken burgers compared to beef burgers or hot dogs [78]. Often, such high levels of TFAs are associated with the high content of them in the foods used in the diet of the animals [77]. Therefore, the TFAs in chicken burgers could be due to the diet the chickens have had, not just in the final product offered for sale to the consumer [78]. Likewise, in milk, the percentages of trans isomers are higher than in meat [77].

In Spain, several reports have demonstrated that the TFAs are present in processed and ultra-processed foods; however, the percentage has been decreasing over the years [79]. The Spanish security agency for food and nutrition (AESAN) established that less than 1% of TFAs, or approximately 2.1 g/day, of the total caloric intake was consumed by 2010 [79]. In 2015, AESAN concluded there had been a very favorable impact of the public policies of previous years, in reducing the use of *trans fats* by the food industry [80]. Prior to the new legislation limiting 2 g of *trans fats* per 100 g in food products, reports showed a voluntary reduction in the use of *trans fats* by the food industry. For this reason, information campaigns have been created for decades by the Ministry of Health in Spain and other institutions to promote good eating habits in the younger population and encourage exercise so that a favorable relationship between food and health is maintained. In this context, to contextualize, in Spain, whole cow’s milk is one of the main foods that contributes lipids to the diet [81]. In addition, the composition of dairy derivatives varies depending on the amounts and type of milk they contain. Ice creams made with milk fats usually contain vegetable fat and present low percentages of trans isomers [77]. However, ice creams made with phTFAs have been found to contain higher levels of TFAs, ranging from 14% to 31% [62,82].

### Replacing Trans Fat in Foods

Different international organizations have issued recommendations and strategies to eliminate or reduce the use of TFAs in food products [70]. One such initiative is the global action plan, “Replace Trans Fat”, launched by the World Health Organization (WHO) in 2018. Its goal is to eliminate industrially produced TFAs from the global food supply by 2023. While progress has been made in many countries towards reducing or eliminating TFAs in food products, compliance with TFA limits may vary [83]. The European Union (EU) has also taken action to reduce TFAs in food products, as reflected in the new Regulation (EC) No. 1925/2006, which cites “Replace Trans Fat” in its considerations section to justify the legislative measures taken by the EU [84]. The “Replace Trans Fat” (REPLACE) initiative has gained international recognition and has been used as a model for other organizations to develop their own action plans [72]. For instance, the Pan American Health Organization (PAHO) published the Plan of Action to Eliminate Industrially Produced *Trans Fats* 2020–2025 [39], which provides member states with strategies to create public policies and legislation on TFA reduction (PAHO, 2020). However, while these initiatives provide a framework for reducing TFAs in food products, ongoing monitoring and evaluation is necessary to ensure compliance and progress towards the goal of eliminating TFAs from the global food supply.

In this respect, the first strategy for TFA replacement should focus on listing products that contain TFAs. Second, promoting their substitution by oils or unsaturated fats which are healthier. The World Health Organization (WHO) recommends legislative action as the third strategy for eliminating *trans fats* (TFAs) [36]. This involves providing guidance for setting standards within the current regulatory framework that either limit or eliminate industrially produced TFAs [64]. This will allow the legal framework to be adapted [85]. The WHO claim that such a measure would be most effective, as voluntary action from the food industry has shown varying levels of commitment [86]. Fourth, to enforce stricter TFA regulations, the content of *trans fats* in industrially produced food and consumption of these fats among the population should be evaluated [87,88]. These evaluations serve the following two purposes: first, to analyze whether the levels established for the use of TFAs are respected by the food industry, and secondly, to determine whether the science in the health sector demonstrates the adverse effects of these quantities [83]. The fifth strategy involves raising awareness among policy-makers, producers, and distributors, as well as the general public, about the harmful effects of trans-fatty acids on health [72]. This is followed by the sixth action proposed by REPLACE, which is to demand compliance with policies and legislation that prohibit or limit *trans fats*. The adoption and implementation of these strategies proposed by REPLACE, including legislative measures, awareness-raising campaigns, and the reformulation of food products, would potentially reduce global consumption of TFAs by up to 75% by 2025 (WHO, 2018) [88].

## 5. Legal Framework of Trans-Fatty Acids

### 5.1. The European Parliament and the Council of the European Union Legal Basis

Regulation (EC) No. 178/2002 is a regulation of the European Parliament and the Council of the European Union that establishes the general principles and requirements of food law and establishes the European Food Safety Authority (EFSA) [89]. This EU regulation aims to ensure a high level of protection of human health and consumer interests in relation to food, while ensuring the effective functioning of the internal market [90]. Further, this instrument sets out the requirements for food businesses to ensure that the food they produce is safe for consumption and meets certain quality standards. It also establishes procedures for risk assessment and risk management in relation to food safety, and requires the traceability of food products throughout the supply chain [91]. In spite of the fact that this EU regulation is important legislation for ensuring food safety and protecting public health, it also presents challenges in terms of compliance and regulatory responsibility [92]. Thus, future perspectives for food safety regulation may need to address these challenges while maintaining a high level of protection for consumers [90].

The definition of food in Regulation (EC) No. 178/2002 agreed with the one described by other international bodies, such as the World Health Organization (WHO) and the Codex Alimentarius Commission. These organizations define food as any substance or product, whether processed, semi-processed, or raw, intended for human consumption [89]. However, it is true that different organizations may have different interpretations of the concept of food and how it should be regulated. For instance, the Pan American Health Organization (PAHO) has identified several categories of products that are commonly considered as food but may have additional functions or purposes beyond nutrition [39]. For instance, some food supplements, herbal products, and functional foods are recognized by PAHO for their potential health benefits. However, these products also require specific regulation to ensure their safety and quality [39]. Similarly, the EU has developed a comprehensive regulatory framework for food that goes beyond the traditional definition of food. Indeed, the EU has established regulations for novel foods, food supplements, and food additives, as well as for non-food products that may come into contact with food, such as packaging materials. Thus, while the definition of food in regulation (EC) No. 178/2002 is widely accepted and used by international bodies, different organizations may have different interpretations and regulatory frameworks based on their specific contexts and objectives. Therefore, it is important to consider the specific regulations and policies of each organization when evaluating the interpretation of the concept of food [89].

### 5.2. Legal Context of Ultra-Processed Food Products

Based on recent research, it has been demonstrated that ultra-processed products containing *trans fats* cannot be considered as healthy food products due to their harmful effects on human health [93,94]. Indeed, *trans fats* increase the levels of LDL cholesterol, contributing to the development of cardiovascular diseases, diabetes, and other health issues among others [95]. Consequently, the WHO has recommended the elimination of *trans fats* from the global food supply by 2023 [71]. In contrast, some fats, such as omega-3 and omega-6 fatty acids, provide beneficial effects on human health, contributing to the prevention of heart disease, diabetes, and other chronic diseases [96]. It is, therefore, important to differentiate between the different types of fats found in food and food products. Table 3 summarizes the most common and consumed ultra-processed foods (UPFs) and their nutritional composition in the EU and Spain. Regarding the types of *trans fats*, there are two categories, the naturally occurring and artificial *trans fats*. Natural *trans fats* can be found in small amounts in some animal products, such as milk and meat [97]. Artificial *trans fats* are produced during the hydrogenation process of vegetable oils, commonly used in industrial food production [63]. Table 4 summarizes the most relevant academic references on the legal framework, trans fats and ultra-processed foods in the European Union, Spain, and their impact on human health.

In the European Union, the use of artificial *trans fats* has been limited since 2007, and in 2019, the EFSA recommended the reduction in the daily intake of *trans fats* to below 1% of total energy intake [33]. Likewise, in Spain, several regulations have been implemented to restrict the use of artificial *trans fats* in food products [102]. Ultra-processed products that contain *trans fats* should not be considered as food products, as they do not provide beneficial nutritional value to consumers [103]. These fats have been associated with the appearance of several chronic diseases and are typically found in partially hydrogenated vegetable oils, which are commonly used in processed and fried foods to enhance the flavor, texture, and shelf life [12,104]. However, as mentioned above, not all types of *trans fats* have the same effects on health. Therefore, it is important to limit consumption of iTFAs by avoiding ultra-processed foods that contain partially hydrogenated vegetable oils. Instead, consumers should choose foods that contain healthy fats, such as monounsaturated and polyunsaturated fats found in nuts, seeds, fish, and vegetable oils [93].

**Table 4:** A summary of the most relevant academic references on legal framework, trans fats, and ultra-processed foods in the European Union, Spain, and their impact on human health.

Key Information	Year	Reference
*Greater UPF consumption has been a key driver of obesity.*	2024	[3]
*Examines NGO governance functions and their patterns of engagement and participation across institutional spheres. Overall, the article makes a twofold contribution to existing debates.*	2024	[105]
*Analysis of legislation and positions on trans-fatty acids presence in food*	2024	[104]
*European Union Legislation Nutrition Food Regulations*	2023	[106]
*Sustainable food systems framework, EU*	2023	[107]
*Food safety; novel foods; risk assessment; Cannabis sativa; tetrahydrocannabinol; food supplements; cannabidiol; benchmark dose; health-based guidance value (HBGV); liver toxicity*	2023	[108]
*Underutilized legumes; fungi; insects; aquatic weeds; bioactive peptides; food formulation; novel foods; alternative proteins*	2023	[109]
*Food handlers; food safety; hygiene; food establishments*	2023	[110]
*Food safety assessment, food contact materials, evaluation.*	2023	[111]
*Food safety, food chain, regulation, environmental safety.*	2023	[112]
*Healthy food environments; policies*	2022	[113]
*Ultra-processed Food Consumption*	2022	[10]
*Consumer Knowledge about Food*	2021	[114]
*European Food Safety Authority; nutrition; functional foods; European food law; risk assessment*	2021	[115]
*Novel foods, EFSA Food Safety assessment, Risk assessment, Nutrition, Whole foods, Mixtures*	2020	[116]
*Risk assessment, food, regulatory, governance*		[117]
*One Health approach to food safety*	2020	[118]
*EFSA, Risk assessment, Risk management, Nutritional sciences, Nutrition*	2019	[119]
*Safety in Wine Production*	2019	[120]
*Consumption of ultra-processed foods and cancer risk*	2018	[121]
*Tofu Steaks? Developments on the Naming andMarketing of Plant-based Foods*	2018	[122]
*Cross-border parcel delivery*	2017	[123]
*Dietary guidelines*	2017	[124]

## 6. Regulation to Limit the Use of *Trans Fats* in the European Union and Spain

The regulation of *trans fats* has its origins in the growing scientific evidence that began to emerge in the mid-20th century. *Trans fats*, primarily produced through the partial hydrogenation of vegetable oils, were initially adopted by the food industry due to their low cost and ability to extend product shelf life [125]. However, subsequent studies showed that these fats increase the risk of cardiovascular diseases by raising LDL (bad) cholesterol and lowering HDL (good) cholesterol. The first country to take significant action was Denmark, which in 2003 established a limit for industrial *trans fats*, setting a maximum of 2 g per 100 g of total fat in foods. This regulation was motivated by the scientific evidence on health risks and had a notable impact on reducing deaths related to cardiovascular diseases in the country [126,127].

At the European level, regulation was slower to develop. Despite recommendations from organizations such as the World Health Organization (WHO) and the European Food Safety Authority (EFSA), only a few countries like Austria, Hungary, and Latvia initially followed Denmark’s example. However, increasing pressure from health organizations and concerned consumers led to broader action. In April 2019, the European Commission adopted a regulation that limits industrial *trans fats* to a maximum of 2 g per 100 g of fat in foods. This regulation came into effect in April 2021, marking an important step towards reducing trans-fat consumption across Europe. The measure aims to align European policies with international best practices and significantly reduce the risk associated with these fats [43].

Also, it is important to mention the TRANSFAIR project, which was an initiative funded by the European Commission in 1995, aimed at investigating the consumption of trans-fatty acids (TFAs) in Western Europe, with a specific focus on these compounds. This project arose from increasing concerns about the adverse health effects of TFAs, particularly regarding cardiovascular diseases. Through TRANSFAIR, significant data were gathered on TFA consumption levels across various EU member states. This comprehensive data collection enabled the European Commission to evaluate and compare dietary patterns among different countries. For instance, countries adhering to a Mediterranean diet exhibited notably lower TFA consumption levels [127]. The project highlighted the variation in TFA intake among European countries, with Mediterranean nations generally showing the lowest levels of consumption. This difference was attributed to dietary habits that favor healthier fats over industrially produced TFAs. The findings from TRANSFAIR contributed to a broader understanding of dietary impacts on health and informed subsequent regulatory measures within the EU to address and limit TFA intake. The European Food Safety Authority (EFSA) has since emphasized the need to minimize TFA consumption due to their negative impact on heart health, which is more pronounced than that of saturated fats. These efforts have been part of a larger movement across Europe to reformulate food products and reduce TFA content, aligning with global health recommendations to mitigate cardiovascular risks [127].

The TRANSFAIR project provided the European Commission with the opportunity to collect data on trans-fatty acid (TFA) consumption in various member states of the European Union. The study results showed that countries following a Mediterranean diet, such as Greece, Italy, and Spain, had lower levels of daily TFA consumption in grams. Although TFA levels in Europe during 1995 and 1996 were not considered dangerous, the EU and its member states continued to promote the reduction in TFA consumption. To achieve this, they established agreements with the food industry to gradually replace *trans fats* in their products [128]. In 2006, the European Food Safety Authority (EFSA) recommended reducing TFA consumption as much as possible. In response to this recommendation, Denmark implemented a regulation that same year limiting TFA content to 2% of total fat in food products, which resulted in an approximate 60% reduction in cardiovascular diseases [129]. Motivated by Denmark’s success, the EU commissioned several reports to expand their understanding of TFAs and develop a legislative action plan to reduce their use. One of these reports, adopted in 2015, highlighted that coronary diseases were a leading cause of death in the EU due to high TFA consumption, which increased the risk of heart disease and other related issues. Over time, with guidance from the World Health Organization (WHO) and the Food and Agriculture Organization (FAO), the EU adopted new safety policies and eventually implemented regulations to reduce TFA use. The specific details of these regulations are addressed in the following sections of the text [126].

### 6.1. Regulations of the European Union (EU)

The regulations of the European Union (EU) on the use of fats and substances in food production has evolved significantly over the past few decades, with a particular focus on *trans fats* due to their negative effects on cardiovascular health. The origin of these regulations dates back to Regulation (EC) No 1925/2006 of the European Parliament and of the Council, enacted on 20 December 2006. This regulation established a framework for the addition of vitamins, minerals, and other substances to foods. Although it did not specifically focus on *trans fats*, it laid the groundwork for future regulations on food additives and potentially harmful substances [130].

In 2009, the European Food Safety Authority (EFSA) issued a crucial scientific opinion on *trans fats*. EFSA concluded that the intake of trans-fatty acids should be as low as possible within the context of a nutritionally adequate diet. This statement marked a turning point in the regulatory perception of *trans fats* in the EU [131]. As scientific evidence on the harmful effects of *trans fats* accumulated, several EU member states began implementing their own regulations. Denmark was a pioneer in this regard, establishing in 2003 a limit of 2 g of *trans fats* per 100 g of fat in foods. Other countries such as Austria, Hungary, and Latvia followed suit in subsequent years.

In December 2015, the European Commission published a crucial report on *trans fats* in foods and in the overall diet of the EU population. This report not only reviewed the current situation but also called for the establishment of a legal limit on *trans fats* at the EU level [132]. The report argued that this measure would be the most effective in protecting public health and ensuring a level playing field for all economic operators. Following the recommendations of this report and in line with World Health Organization (WHO) guidelines, which urged the elimination of industrially produced *trans fats* from the global food supply by 2023, the EU began working on stricter regulation. The result of these efforts was the adoption of Commission Regulation (EU) 2019/649 on 24 April 2019. This regulation amended Annex III to Regulation (EC) No 1925/2006, establishing strict limits on the amount of industrially produced *trans fats* in all foods sold to EU consumers [11].

**Figure 2:**
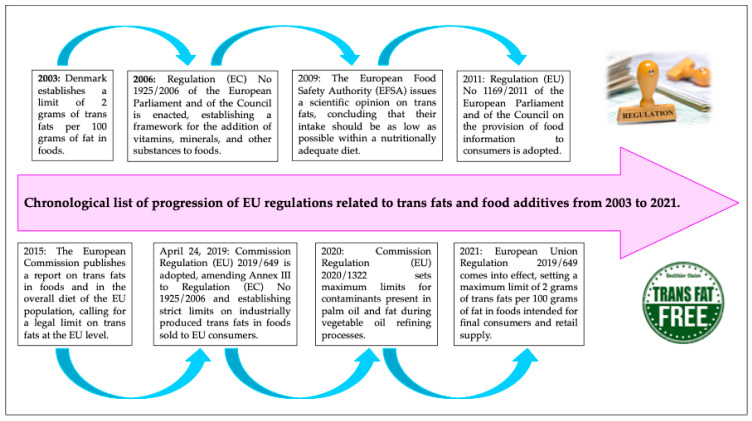
Chronological list on progression of regulations related to *trans fats* and food additives from 2003 to 2021.

The European Union Regulation 2019/649, which came into effect on 1 April 2021, establishes key points for regulating the trans fat content in food products. This regulation sets a maximum limit of 2 g of *trans fats* per 100 g of fat in foods intended for final consumers and retail supply [11]. It is important to note that this limitation applies exclusively to industrially produced *trans fats*, excluding those naturally occurring in fats of animal origin. Establishing this legal limit is considered the most effective way of protecting consumers from coronary diseases, the leading cause of death in the EU, linked to high levels of trans and saturated fatty acids. According to Article 2 of the regulation, food industry operators supplying products intended for final consumption or retailers must ensure they provide information on the trans-fat content exceeding 2 g per 100 g of fat, excluding naturally occurring *trans fats* in animal-origin fats. If AGT-PI levels are below this threshold, such information is not required. The EU has missed an opportunity to further limit the use of *trans fats* by the food industry and improve labeling regulations. It is hoped that future regulations will require clear and precise information on the trans fat content in each food product. Although labeling *trans fats* is not mandatory, the EU has set limits on their use and restrictions on quantities used in food production. Specifically, palm oil and fat have maximum limits established by Commission Regulation (EU) 2020/1322 due to contaminants present during vegetable oil refining processes. These limits do not apply to virgin oils like olive oil. In Figure 2, the chronological list on the progression of regulations related to *trans fats* and food additives from 2003 to 2021 is shown. In the present days, the implementation of this regulation represents a significant challenge for the food industry.

At this point, it is necessary to note briefly that the formation of trans-fatty acids during the oil refining process, specifically in the deodorization stage, occurs as a result of a steam distillation process under specific conditions of high vacuum and elevated temperatures (220–260 °C). During this process, undesirable substances such as free fatty acids, odors, flavors, and other volatile components are removed, but simultaneously trans-fatty acids can form due to the geometric isomerization of unsaturated fatty acids [133,134].

In this context, FoodDrinkEurope, the organization representing the EU food industry, welcomed the adoption of the regulation and committed to supporting companies that might face technological challenges in complying with the new standards. It is important to note that this regulation aligns with the WHO recommendations and represents a significant step in harmonizing food standards across the EU. Additionally, it contributes to public health protection by addressing the risks associated with trans-fat consumption, which has been linked to an increased risk of cardiovascular diseases. The regulation has also had an impact on food labeling. Although it does not specifically require *trans fats* to be labeled on food products, the limitation on their use has led many manufacturers to reformulate their products and, in some cases, to promote the absence of *trans fats* as a health benefit.

### 6.2. Regulation in Spain

The regulation of trans-fatty acids in Spain has developed gradually, aligning with European guidelines and international health recommendations. The main legislative framework is established in Law 17/2011 on Food Safety and Nutrition, enacted on 5 July 2011. This law, in its Article 43, sets out specific obligations for food industry operators regarding TFAs [135]. According to this law, operators must establish adequate conditions in their industrial processes to minimize the formation of TFAs when these are intended for food consumption. Additionally, they are required to demand information from their suppliers about the TFA content in the foods or raw materials supplied to them. This information must be available to the Administration when requested. It is important to note that these requirements do not apply to animal products that naturally contain TFAs [135,136,137,138].

Previously, Royal Decree 867/2008, of 23 May, had already established specific limits for TFA content in infant formulas. This decree approves the specific technical–sanitary regulations for infant formulas and follow-on formulas, aligning with the European Union Directive 2006/141/EC [136,138]. In the educational sphere, Article 40.6 of Law 17/2011 prohibits the sale of foods and beverages with high content of saturated fatty acids, trans-fatty acids, salt, and sugars in nursery schools and educational centers [135,137]. However, the law does not specify exact limits, indicating that these should be established by regulation. Spain, as a member of the European Union, is also governed by EU regulations.

**Figure 3:**
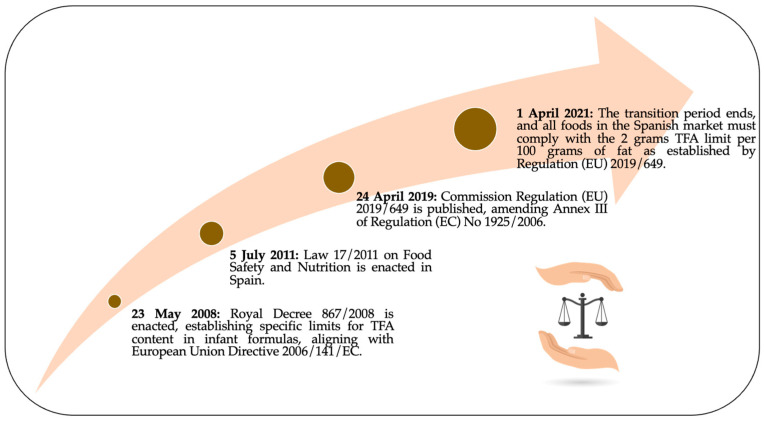
Chronological list on the progression of regulations related to trans-fatty acids in Spain from 2008 to 2021, including national and EU regulations that apply to Spain as a member state.

In this context, Commission Regulation (EU) 2019/649, published on 24 April 2019, marks an important milestone. This regulation amends Annex III of Regulation (EC) No 1925/2006 and establishes a maximum limit of 2 g of TFAs per 100 g of fat in foods intended for the final consumer and retail supply. It is crucial to note that this limit applies only to industrially produced TFAs, excluding those naturally present in fats of animal origin. Regulation 2019/649 came into force on 15 May 2019, but allowed a transition period until 1 April 2021, for the food industry to adapt. From this date, all foods in the Spanish market must comply with this limit. In addition to setting limits, the regulation also imposes information obligations between operators. When foods are not intended for the final consumer or retail supply, operators must provide information on the amount of TFAs if it exceeds the established limit, thus ensuring compliance in the final product. Figure 3 shows the chronological list of the progression of regulations related to trans-fatty acids in Spain from 2008 to 2021, including national and EU regulations that apply to Spain as a member state [136,137,138].

The Spanish Agency for Food Safety and Nutrition (AESAN) has played a crucial role in the implementation and monitoring of these regulations. In 2015, AESAN conducted a study to evaluate the TFA content in foods in Spain, comparing the results with a previous study from 2010. The findings showed that most of the analyzed food groups had a TFA content and percentage below 2% of total fat, indicating a decreasing trend in TFA levels. Despite these advances, some experts consider that Spanish regulation could be stricter, especially regarding the specific labeling of TFAs and the application of limits to all types of foods, including those sold in vending machines.

### 6.3. Nutritional Labeling Regulations and Deficiencies: Spanish and European Directives and Initiatives

In accordance with the provisions of the Codex Alimentarius (EU), nutritional labeling must be clear and specific to provide consumers with information about food so they can make informed choices that contribute to healthy eating habits and improvements in population health. For solid food products, it is allowed to indicate that the product is “low in saturated fat” when the total amount of saturated and *trans fats* in the product is less than 1.5 g per 100 g of product. For liquid food products, they may also indicate that they are “low in saturated fat” when the total amount of saturated and *trans fats* in the product is less than 0.75 mL per 100 mL.

Some food labels show the total energy contribution of the food, and the part corresponding to *trans fats* plus saturated fats should be less than 10%. In Spain, RD 2012/1992 of 6 March approves the general standard for labeling, presentation, and advertising of food products. It was modified by RD 930/1995 of 9 June to incorporate Directive 79/112/CEE into the Spanish legal system. Directives 94/54/CE and 96/21/CE were adopted to complement this Directive. They mention the obligation to provide certain information on labeling. These Directives were incorporated into the Spanish legal system through RD 1908/1995 of 24 November and RD 1268/1997 of 24 July.

RD 1334/1999 incorporated the Directive of the European Parliament and Council 97/4/CE of 27 January, which made it mandatory for the first time to list the quantities of some ingredients on the label. Article 7 specifies that the list of ingredients should be preceded by the title “ingredients”, and the ingredients should be listed in descending order of their weight when they were incorporated into the product. In relation to refined fats, partially hydrogenated fats, or *trans fats*, it is not required to specify them on the labeling, as long as the total fat content is indicated. However, Annex I specifies that refined fats should be designated in the labeling with the qualifier of their “vegetable” or “animal” origin, such as “sunflower oil”, and when hydrogenated fats are used, this must be mentioned. It is necessary to identify the specific type and amount of hydrogenated fats in the product to avoid misleading consumers who may confuse saturated fats with *trans fats*. Overall, the regulations on food labeling could benefit from greater clarity and specificity to ensure that consumers can make informed choices about the food they consume.

Regulation (EU) No. 1169/2011 of the European Parliament and of the Council, adopted on 25 November 2011, is a pivotal piece of legislation concerning the provision of food information to consumers within the European Union. Although it was enacted in 2011, its enforcement began in January 2014. The regulation mandates that labels on most food products include essential nutritional information such as energy value, fat content, saturated fatty acids, carbohydrates, sugars, protein, and salt. This is designed to ensure that consumers have access to clear and comprehensive information to make informed dietary choices. In addition to these mandatory elements, the regulation allows for voluntary supplementary information. For instance, labels can include details about the amounts of *trans fats* or mono- and polyunsaturated fatty acids, provided such claims are scientifically substantiated. Some products, like the ProActiv brand margarine, already break down fat types into categories such as monounsaturated, polyunsaturated, and saturated fats. The regulation aims to standardize food labeling across EU member states, enhancing transparency and consumer protection. It applies to all stages of the food supply chain where consumer information is relevant. This comprehensive approach not only facilitates informed consumer choices but also aligns with broader public health goals by encouraging healthier eating habits through improved transparency in food labeling.

Since Regulation No. 1169/2011 came into force, palm oil must be included in the list of ingredients in a mandatory manner because the Regulation requires the specific plant origin to be designated. Based on our analysis, we have concluded that food labeling is deficient and it is necessary to take into account the labeling proposals by PAHO that are increasing in America, such as using black hexagons with white lettering that are eye-catching and easily visible, which make it possible to detect whether packaged products contain high amounts of fat, salt, or sugar, among other compounds.

Similarly, the strategy followed in Canada since 2003 should be appreciated as a reference, as this country requires that the content of AGT must appear on the food label. Following the example of their neighboring country, the United States has required since 2006 that the TFA content is indicated on the label, which should be provided on a separate line from the nutritional values and its components, so that it is easier for consumers to read this information [FESNAD 2013].

However, in Spain, most manufacturers do not publicly disclose the amounts of AGT-PI contained in their food products, but they introduce advertising that can be perceived as a product with nutritional advantage and encourage its selection over the competition. A significant example is in the Hacendado brand of lasagnas, specifically the Bolognese lasagna, which is a 1.1 kg quick-frozen pre-cooked food. At the end of the ingredients list on the back of the product package, it indicates “without hydrogenated fats” and “without dyes”. However, the product may still contain hydrogenated fats, even if they are of natural origin, because lasagna contains milk and beef. This can lead consumers to make decisions that are not based on fully truthful information. Therefore, the label should have stated “partially fat-free” or “without industrial hydrogenated fats” to provide accurate information to consumers. Table 5 shows the chronological list describes the progression of food labeling and trans-fat regulations in Spain, the EU, and North America from 1992 to 2021.

## 7. Discussion

The presence of industrial *trans fats* in processed and ultra-processed food products represents a significant challenge at the intersection of public health, food regulation, and consumer rights. The complexity of this issue has deepened with recent global events and evolving consumer awareness, necessitating a more comprehensive analysis of its various dimensions.

The analysis of the theoretical implications reveals significant contradictions in the current regulatory framework of the European Union and Spain. While strict regulations on *trans fats* exist, such as Law 17/2011 and Directive 1999/21/EC, there remains a notable gap between regulatory theory and industrial practice. This disconnect is particularly evident in the marketing of ultra-processed foods as “healthy” or “low-fat”, even when they contain *trans fats*. The persistence of this paradox raises questions about the effectiveness of current regulatory mechanisms and the need for more stringent enforcement measures.

The war between Russia and Ukraine has exposed critical vulnerabilities in food supply chains, directly affecting the nutritional quality of products. The observed increase in the use of refined oils and *trans fats* as economical alternatives during this crisis demonstrates how geopolitical factors can significantly impact public health. This situation has highlighted the need for more resilient food systems and alternative sourcing strategies. Furthermore, the economic pressures resulting from the conflict have led some manufacturers to compromise on ingredient quality, potentially increasing the prevalence of harmful substances in food products.

From a practical perspective, strengthening the monitoring and control systems in the food industry has become increasingly urgent. This requires implementing systematic sampling programs across different regions, developing more efficient methods for detecting *trans fats*, and establishing stricter sanctions for non-compliance. The current testing methodologies, while effective, often face challenges in terms of cost and implementation speed, suggesting the need for innovative approaches to food safety monitoring.

The food industry must undergo significant transformation, prioritizing product reformulation and innovation. This includes replacing *trans fats* with healthier alternatives, investing in research to develop new processing technologies, and improving transparency in nutritional labeling. However, these changes present technical and economic challenges that need to be addressed through collaborative efforts between industry stakeholders, research institutions, and regulatory bodies.

The legal framework requires comprehensive updating to integrate the right to healthy food as a fundamental right. This involves establishing stricter limits for *trans fats* in processed products and improving coordination between national and international regulatory bodies. The current fragmentation of regulatory oversight has created loopholes that some manufacturers exploit, necessitating a more unified approach to food safety governance.

Consumer protection initiatives must evolve beyond basic labeling requirements. Educational programs on nutrition and label reading need to be complemented with early warning systems for non-compliant products and effective mechanisms for citizen participation in food policy formulation. The rise of digital technologies offers new opportunities for consumer engagement and transparency in food safety monitoring.

The experience with programs such as NAOS and PERSEO has revealed both successes and limitations in the current approaches to trans-fat regulation. These programs have demonstrated the need for more robust and enforceable measures, particularly in addressing industry compliance and consumer awareness. Success in reducing *trans fats* will depend on aligning food industry interests with public health objectives, especially during economic crises and global conflicts. The effective implementation of these measures requires sustained commitment from both regulatory authorities and the food industry, supported by active participation from civil society and the scientific community. This multi-stakeholder approach should focus on developing innovative solutions while ensuring economic viability for food manufacturers.

Looking forward, the effective regulation of *trans fats* demands a multidimensional approach that considers economic, social, and public health aspects. The strategies must address not only the technical aspects of food production but also the broader socioeconomic implications of regulatory measures. This includes considering the impact on small and medium-sized food producers, who may face greater challenges in implementing required changes.

Continued research and monitoring of policy implementation will be crucial for ensuring the effectiveness and sustainability of adopted measures. This includes studying the long-term health impacts of trans-fat alternatives, evaluating the economic feasibility of proposed changes, and assessing the effectiveness of different regulatory approaches across various contexts. The protection of public health and consumer rights in the current food context requires ongoing adaptation to emerging challenges. This includes addressing new forms of food processing, changing consumer preferences, and evolving global supply chain dynamics. Success in this area will require sustained collaboration between all stakeholders and a commitment to evidence-based policy making.

## 8. Future Research Directions

According to the revised literature presented in this work, there are a few future research directions that deserve special attention and are highlighted as follows:

Investigating the effectiveness of current regulations: To date, there are regulations in place to limit the use of *trans fats* in the food industry; it is important to assess the effectiveness of these regulations in reducing the consumption of *trans fats* and improving public health. Research can evaluate how well food manufacturers are complying with these regulations and whether consumers are aware of the regulations and the potential risks of consuming *trans fats*.

Studying the impact of alternative fats: Food manufacturers are replacing *trans fats* with healthier alternatives, such as unsaturated fats or saturated fats with a shorter chain length. It is important to assess the impact of these alternatives on the nutritional content and health effects of the food products. This research can help identify the most effective alternatives to *trans fats* and inform future regulations and guidelines.

Examining the labeling of ultra-processed foods: As mentioned in the discussion, there are contradictions in the legal context of ultra-processed food products, as they are often labeled as “healthy” or “low-fat”, while still containing *trans fats*. Research can evaluate the accuracy and completeness of the labeling of ultra-processed foods, including the disclosure of trans-fat content, and identify ways to improve transparency and clarity for consumers.

Investigating the long-term health effects of *trans fats*: Despite the negative impact of *trans fats* on health having been established, more research is needed to understand the long-term health effects of consuming *trans fats*, especially at low levels. This research can inform the development of guidelines and recommendations for safe levels of trans-fat consumption.

Exploring the impact of cultural and social factors: Cultural and social factors can influence the consumption of *trans fats*, such as food preferences, habits, and traditions. Research can examine these factors and their impact on the consumption of *trans fats* and inform the development of culturally sensitive and effective interventions to reduce the consumption of *trans fats*.

In addition to the previously discussed future research directions, the presented analysis of current EU and Spanish regulations on iTFAs, particularly EU Regulation 2019/649 and Spain’s Law 17/2011, suggests the two following additional key areas for investigation: First, developing more standardized measurement protocols for iTFA content, addressing the methodological challenges identified in the present work. Second, investigating the impact of recent regulatory changes on consumer awareness and industry practices, particularly in relation to labeling requirements under EU Regulation 1169/2011. These directions emerge from the gaps and challenges identified in the comprehensive analysis of the legal framework and health implications of iTFAs in the EU and Spain [1,10,11,42,43,130].

## 9. Conclusions

Throughout this research, it has been identified that industrial trans-fatty acids (iTFAs) represent a significant challenge to public health in the European Union and Spain. Scientific evidence has clearly demonstrated that the consumption of iTFAs is associated with an increased risk of cardiovascular diseases, type 2 diabetes, and certain types of cancer. Even a small increase in daily iTFA intake can have serious health consequences, with studies showing that a 2% increase in energy intake from iTFAs can increase the risk of cardiovascular disease by 23%. In response to these risks, both the EU and Spain have implemented regulations to limit the use of iTFAs in food, such as Regulation (EU) No 1169/2011 and Law 17/2011. However, significant challenges persist in the effective application of these regulations. The lack of uniform compliance among member countries and difficulties in detecting iTFAs in food complicate efforts to protect consumers. The increase in consumption of ultra-processed foods, which often contain high levels of iTFAs, is particularly concerning. In some European countries, including Spain, these products can represent up to 50% of total daily energy intake. This trend underscores the urgent need for stricter and more effective policies to reduce the use of iTFAs in the food industry. The World Health Organization recommends that trans-fat intake should not exceed 1% of total daily calories. To achieve this goal, it is crucial to adopt a multifaceted approach that includes strengthening and harmonizing existing regulations throughout the EU, improving monitoring and detection systems for iTFAs in processed foods, encouraging the reformulation of food products to use healthier alternatives, implementing comprehensive educational campaigns to inform consumers about the risks associated with iTFA consumption, and promoting ongoing research on the long-term effects of iTFAs and best practices for their elimination in the food industry. The effective elimination of iTFAs from the European food chain will require a collaborative effort among legislators, the food industry, scientists, and consumers. Only through this holistic approach can public health be adequately protected and a healthier and safer food supply be ensured for all European citizens.

Further, the present work has provided a comprehensive analysis of iTFA regulations and health implications in the EU and Spain, while acknowledging certain methodological considerations that should be considered. Indeed, the rapid evolution of iTFA regulations, particularly following the implementation of EU Regulation 2019/649 and Spain’s Law 17/2011, means that some recent regulatory changes may not be fully reflected in the current literature. Additionally, while the present analysis of the legal framework is extensive, access to industry data regarding actual iTFA content in food products remains limited, as some information is considered proprietary. These limitations highlight the ongoing need for more transparent reporting mechanisms and standardized measurement protocols across the EU.

## Figures and Tables

**Table 1:** Distribution of the most relevant reviewed literature by subject area.

Subject Area	Number of Articles	Percentage
Legal Framework	34	34.7%
Health Impact Studies	28	28.6%
Food Science	21	21.4%
Regulatory Policy	15	15.3%
Journal Name	Impact Factor	Articles Used
Food Chemistry	7.514	12
European Food Research	4.072	9
Food Policy	4.189	8
Public Health Nutrition	3.242	7
Food Control	5.548	6

**Table 3:** Most consumed ultra-processed foods (UPFs) and their nutritional composition in the EU and Spain.

Ultra-Processed Food Product	Saturated Fatty Acids (g)	Unsaturated Fats (g)	Trans Fats (g)	% of Total Energy Intake (EU)	% of Total Energy Intake (Spain)	References
*Bread*	0.10	0.04	0.01	1.6	1.8	[98]
*Cookies*	4.00	4.00	0.20	3.2	2.6	[42]
*Breakfast Cereals*	0.50	0.50	0.10	1.5	1.2	[99]
*Margarine*	20.00	34.00	0.20	0.5	0.6	[100]
*Chocolate Bars*	10.00	17.00	0.10	1.1	0.8	[101]

**Table 5:** The chronological list describes the progression of food labeling and trans-fat regulations in Spain, the EU, and North America from 1992 to 2021.

Year	Regulation
1992	*Royal Decree 2012/1992 of 6 March approves the General Standard for labeling, presentation, and advertising of food products in Spain.*
1995	*Royal Decree 930/1995 of 9 June modifies RD 2012/1992 to incorporate Directive 79/112/CEE into Spanish law.*
1995	*Royal Decree 1908/1995 of 24 November incorporates Directive 94/54/CE into Spanish law.*
1997	*Royal Decree 1268/1997 of 24 July incorporates Directive 96/21/CE into Spanish law.*
1999	*Royal Decree 1334/1999 incorporates Directive 97/4/CE, making it mandatory to list quantities of some ingredients on labels.*
2003	*Canada requires AGT (trans fat) content to appear on food labels.*
2006	*The United States requires TFA (trans-fatty acid) content to be indicated on food labels.*
2011	*Regulation (EU) No. 1169/2011 is adopted on 25 November, concerning the provision of food information to consumers in the EU.*
2014	*Enforcement of Regulation (EU) No. 1169/2011 begins in January.*
2019	*Commission Regulation (EU) 2019/649 of 24 April amends Annex III to Regulation (EC) No 1925/2006, setting limits on trans fats in foods.*
2021	*As of 1 April, the EU regulation limiting trans fats in foods to 2 g per 100 g of fat comes into full effect.*

## Data Availability

The original contributions presented in the study are included in the article, further inquiries can be directed to the corresponding authors.
